# Familial risks of ovarian cancer by age at diagnosis, proband type and histology

**DOI:** 10.1371/journal.pone.0205000

**Published:** 2018-10-03

**Authors:** Guoqiao Zheng, Hongyao Yu, Anna Kanerva, Asta Försti, Kristina Sundquist, Kari Hemminki

**Affiliations:** 1 Division of Molecular Genetic Epidemiology, German Cancer Research Center (DKFZ), Heidelberg, Germany; 2 Cancer Gene Therapy Group, Faculty of Medicine, University of Helsinki, Helsinki, Finland; 3 Department of Obstetrics and Gynecology, Helsinki University Hospital, Helsinki, Finland; 4 Center for Primary Health Care Research, Lund University, Malmö, Sweden; University of Toronto, CANADA

## Abstract

Ovarian cancer is a heterogeneous disease. Data regarding familial risks for specific proband, age at diagnosis and histology are limited. Such data can assist genetic counseling and help elucidate etiologic differences among various histologic types of ovarian malignancies. By using the Swedish Family-Cancer Database, we calculated relative risks (RRs) for detailed family histories using a two-way comparison, which implied e.g. estimation of RRs for overall ovarian cancer when family history was histology-specific ovarian cancer, and conversely, RRs for histology-specific ovarian cancer when family history was overall ovarian cancer. In families of only mother, only sisters or both mother and sisters diagnosed with ovarian cancer, cancer risks for ovary were 2.40, 2.59 and 10.40, respectively; and were higher for cases diagnosed before the age of 50 years. All histological types showed a familial risk in two-way analyses, except mucinous and sex cord-stromal tumors. RRs for concordant histology were found for serous (2.47), endometrioid (3.59) and mucinous ovarian cancers (6.91). Concordant familial risks were highest for mucinous cancer; for others, some discordant associations, such as endometrioid-undifferentiated (9.27) and serous-undifferentiated (4.80), showed the highest RRs. Familial risks are high for early-onset patients and for those with multiple affected relatives. Sharing of different histological types of ovarian cancer is likely an indication of the complexity of the underlying mechanisms.

## Introduction

Ovarian cancer is the seventh most common cancer and the eighth leading cause of cancer-related deaths in women worldwide [1]. The incidence is highest in Eastern and Northern Europe. In Sweden, the incidence has been declining during the last decade [2]. Ovarian cancer is a heterogeneous disease; the most common types are epithelial ovarian cancers and they have been divided into two groups (type I and II) based on distinctive clinicopathologic and molecular genetic features [3]. Type I group includes low-grade serous, low-grade endometrioid, clear cell and mucinous carcinomas, which are indolent and have a good prognosis. While in type II group, tumors are more aggressive and are composed of high-grade serous carcinoma, high- grade endometrioid carcinoma, carcinosarcomas and undifferentiated carcinomas. Non-epithelial ovarian malignancies are far less common and contain sex cord-stromal malignancy; the latter includes thecoma, which is the most prevalent non-epithelial ovarian cancer.

Ovarian cancer risk increases with aging and peaks between the ages of 50 and 80 years [4]. In the general Swedish population, the lifetime risk of ovarian cancer is 1% [2]. Reproductive and menstrual factors are strongly influential regarding ovarian cancer. Factors that can decrease the total number of ovulatory cycles such as pregnancy, breastfeeding and use of oral contraceptives reduce the risk for ovarian cancer. Factors that prolong exposure to ovulation, such as low parity, early menarche and late menopause increase the risk for ovarian cancer [4]. Oral contraceptive use is a confirmed protective factor for ovarian cancer and the widespread use of it during recent decades is considered to be one of the main reasons for the decreasing incidence of ovarian cancer [5]. However, an increased risk for mucinous ovarian cancer was observed in individuals using oral contraceptives [6–8]. The protective effect of high parity has also been confirmed and it is most strongly associated with endometrioid and clear cell types [7]. Other non-reproductive factors, which can influence the risk of ovarian cancer, include smoking and body size (height or body mass index) [9, 10].

Family history is a strong risk factor for ovarian cancer, and the relative risk is estimated to be 2.0 to 4.0 for those that have a first-degree relative affected by the disease [11–15]. The siblings’ risks by age difference are similar, which suggests that the familial clustering of ovarian cancer is mainly heritable [16]. Familial risk is associated with mutations in *BRCA1* and *BRCA2* genes, which conferred respectively 59% and 16.5% risk of developing ovarian cancer by the age of 70 in the Epidemiological Study of *BRCA1* and *BRCA2* mutation carriers (EMBRACE) in the UK [17]. Ovarian cancer is also a manifestation in hereditary nonpolyposis colorectal cancer (HNPCC) syndrome which is caused by mutations in *mismatch repair (MMR)* genes [18]. The related lifetime risk of developing epithelial ovarian cancer is around 12% [19]. Mutations in other genes such as *BRIP1*, *RAD51C* and *RAD51D* also contribute to hereditary ovarian cancer [20]. Each histological type of ovarian cancer harbors distinct mutations. Germline alterations of *BRCA1* and *BRCA2* were reported to be associated with high-grade serous histology [21, 22], and families with HNPCC syndrome present a tendency towards endometrioid and clear cell types [19, 23, 24].

Based on the above, it can be hypothesized that histology-specific etiology may exist in ovarian cancers. There are limited data regarding familial risk for specific histologic types of ovarian malignancy; such data may help elucidate etiologic differences among the various histologic types of ovarian malignancy and assist clinical genetic counseling. In this study, we use the recent national Swedish Family-Cancer Database, which included 16.1 million individuals, to estimate the familial risks of ovarian cancer by age at diagnosis, proband type (mother or sisters) and histology.

## Methods

The Swedish Family-Cancer Database (FCD) includes all people born in Sweden since 1932 (offspring generation) together with their biological parents (parental generation) [25]. The latest version of FCD contains 16.1 million individuals among which nearly 2.0 million were cancer patients recorded to the end of 2015. The 3-digital codes of the 7th revision of the International Classification of Diseases (ICD-7) were used to identify ovarian cancers. Histological types of ovarian cancers were classified according to Systemized Nomenclature of Medicine (SNOMED) codes since 1993. The follow-up for cancer in offspring generation (8.8 million individuals) commenced from the beginning of 1958 (for histological analysis it was started in 1993), the birth year, or the immigration year, whichever came latest. The follow-up was terminated when a person was diagnosed with cancer, emigrated or died, or at the end of 2015, whichever came first.

In this study, all the incident cases of ovarian cancers reported between 1958 and 2015 were analyzed. The world standard population was used to calculate age-standardized incidence [26]. For familial risk analysis by proband type, first-degree relatives (including parents and/or siblings), who were affected by ovarian cancers, were considered as family history. However, in the present study only mother-sister, sister-sister and mother-two sisters family history were taken into consideration. Familial risk for individuals diagnosed < 50 years and ≥ 50 years old were estimated separately. A two-way comparison was employed to estimate relative risks (RRs) for overall (histology-specific) ovarian cancer when family history was histology-specific ovarian cancer, and conversely, RRs for histology-specific ovarian cancer when family history was overall (histology-specific) ovarian cancer. The reference group was individuals without a family history of ovarian cancer, i.e. unaffected relatives.

Poisson regression model was employed to estimate RRs and corresponding confidence intervals (CIs) for 5%, 1% and 0.1% significance levels. Trend tests were performed by modeling the three proband types (only mother, only sisters and mother and sisters) as a continuous covariate. Potential confounders, including age group, calendar period, residential area and socioeconomic status as well as parity were added to the model as covariates. SAS version 9.4 was used to perform the statistical analysis.

The study was approved by the Ethical Committee of Lund University.

## Results and discussion

**Fig 1:**
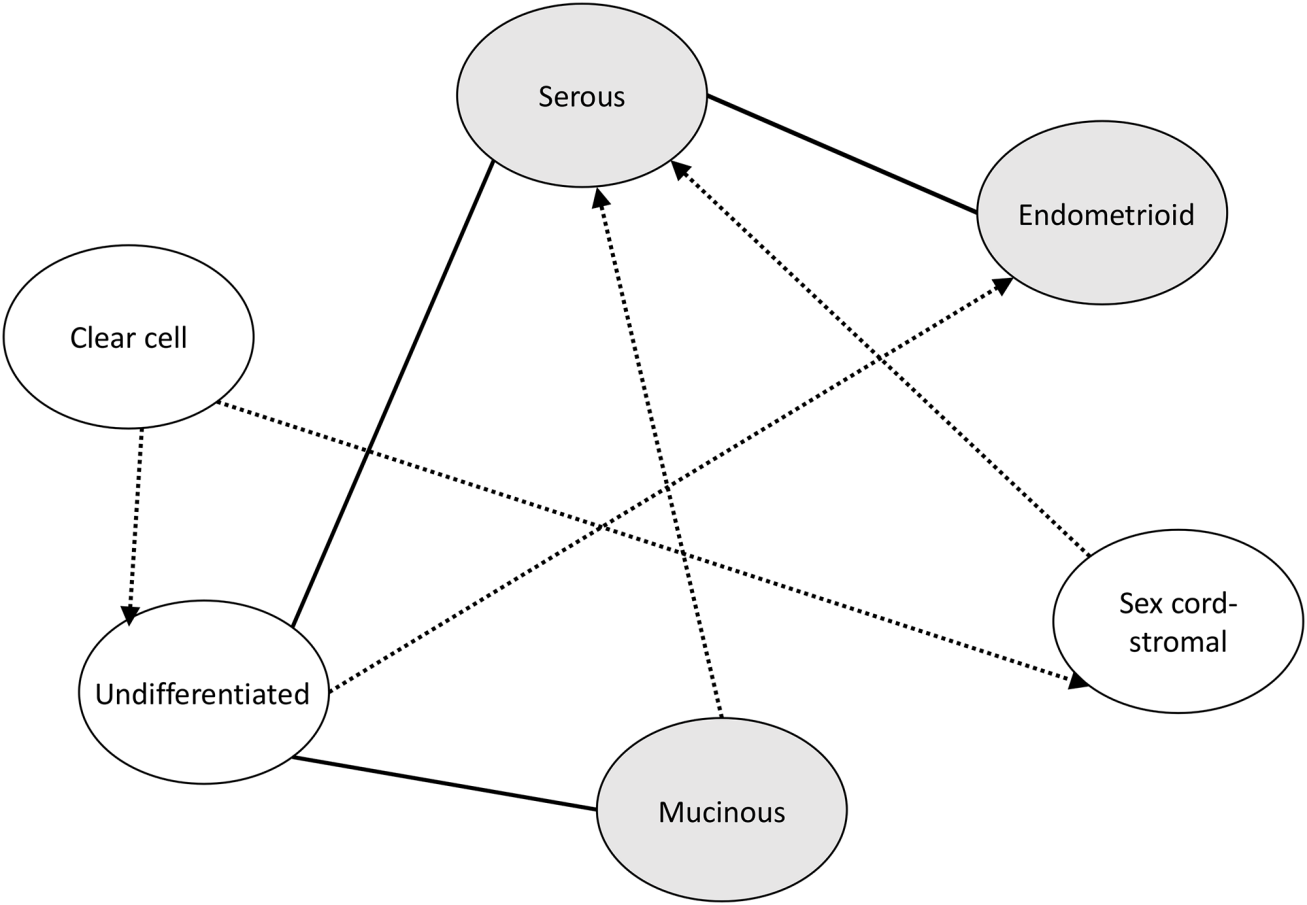
Age-standardized incidence in different time period for overall and eight histological types of invasive ovarian cancer. Since the record of SNOMED was started in 1993, the periods for subtypes only included 1993–2000, 2001–2010 and 2011–2015.

A total of 46,015 ovarian cancer cases were found in our database and of these 11,301 cases were in the offspring generation diagnosed at age 0–83 years, for which RRs were calculated. The age-standardized incidence per 100 000 person-years (Fig 1) in the six periods were the following: 12.6 (1958–1970), 14.7 (1971–1980), 13.4 (1981–1990), 11.2 (1991–2000), 8.9 (2001–2010) and 7.3 (2011–2015). Since the 1970s, the incidence of invasive ovarian cancer has declined in Sweden for a number of reasons, mainly due to the widespread use of oral contraceptives [27]. As for histological type, apart from sex cord-stromal ovarian cancer, incidence of all the other types has decreased. In 2011–2015, the highest incidence was noted for serous (4.23) and endometriod (0.65) types. It has been reported that oral contraceptive use may increase the risk of mucinous ovarian cancer, as opposed to other histologies [6]. Yet the incidence of mucinous ovarian cancer still decreased during 1993–2015, which suggests complex influence of many factors.

The median ages at diagnosis for all ovarian cancers were 63 for the period 1958–2015 and 65 for the period 1993–2015. Ages at diagnosis and proportion of different histological types are displayed in Table 1. Sex cord-stromal type had the lowest age at diagnosis and median ages at diagnosis of other types were all over 60. Age-specific incidence data during 1993 to 2015 are shown in S1 Fig. The maximal incidences for overall and the most common histology-specific ovarian cancers were in the group 70–74 years, which is similar to a Danish report of the period 1978–2002 [28], but relatively younger than the report from the USA in 2011 and older than the report from South Korea during 1999–2012 [29, 30].

**Table 1:** Age at diagnosis in overall and different histological types of ovarian cancer.

Histological types*	Number of cases (%)	Age at diagnosis Median (Q1, Q3)
All^a^	46015	63 (53, 72)
All^b^	16273 (100)	65 (55, 74)
Undifferentiated	411 (2.5)	65 (57, 75)
Clear cell	769 (4.7)	61 (52, 70)
Endometrioid	1760 (10.8)	62 (52, 72)
Serous	7404 (45.5)	65 (57, 74)
Mucinous	1269 (7.8)	62 (50, 73)
Sex cord-stromal ^c^	457 (2.8)	56 (44, 67)
Others ^d^	4203 (25.8)	66 (55, 77)

* All the ovarian cancer cases with histological type information were diagnosed in the period 1993–2015.

^a^: Ovarian cancer cases diagnosed in the period 1958–2015.

^b^: Ovarian cancer cases diagnosed in the period 1993–2015.

^c^: Sex cord-stromal was represented by thecoma.

^d^: Others include histological types of other ovarian cancers such as papillary and germ cell ovarian cancers as well as unspecified ovarian cancers

Serous ovarian cancer accounted for the 45.50% of all the ovarian malignancies, followed by endometrioid type (10.82%). The two proportions are slightly different from the results of the ovarian cancer patients diagnosed during 1993–1990, showing 38.4% for serous and 12.4% for endometrioid types [31]. The changes may be due to the influence of the use of molecular markers for subtypes to regroup some high-grade endometrioid cancer with high-grade serous cancer [32]. Alteration is also observed for the mucinous type, decreasing from 9.7% to 7.8% [31], probably resulted from the application of immunohistochemical staining that can distinguish the primary mucinous ovarian cancer from the metastatic gastrointestinal malignancies [32].

The total number of familial cases was 807 among daughters and mothers; among them a total of 487 were daughters. Thus 4.3% (487/11,301) of invasive ovarian cancer cases were familial in Sweden. The overall familial risk of ovarian cancer was 2.51 (2.29–2.75, *P* <0.001). Table 2 shows that in families of only mother, only sister and both mother and sisters diagnosed with ovarian cancer, familial risks were 2.40, 2.59 and 10.40, respectively, and all of them were significant at the 0.001 level. Risk was higher with a sororal family history, compared to maternal family history, implying recessive inheritance or shared environmental factors among sisters. When considering the cases diagnosed before the age of 50 years the risks increased up to 2.74, 3.86 and 16.05, respectively (*P* <0.001 for all). While for cases over 50 years old, the respective familial risks were lower, 2.22, 2.12 and 8.33, but they were still highly significant (*P* <0.001). Notably, the risks for mother and sister history were equal in the older age group, in contrast to the younger counterpart, which indicates that the excess sororal risk only influenced early onset cases with possible interactions with sex-related hormone levels.

**Table 2:** Familial risk of ovarian cancer in daughters by proband type and age of diagnosis.

Age	Cases without family history	Only mother			Only sisters			Mother and sister			
		*N*	*RR*	*95%CI*	*N*	*RR*	*95%CI*	*N*	*RR*	*95%CI*	*P-trend*
< 50	3872	120	***2*.*74***	2.29–3.26	63	***3*.*86***	3.01–4.96	6	***16*.*05***	7.20–35.74	< .0001
≥50	6942	195	***2*.*22***	1.93–2.56	95	***2*.*12***	1.73–2.60	8	***8*.*33***	4.16–16.67	< .0001
All	10814	315	***2*.*40***	2.14–2.68	158	***2*.*59***	2.21–3.03	14	***10*.*40***	6.16–17.57	< .0001

Bolding, italic and underlining indicate that the 95% CI, 99% CI and 99.9% CI did not overlap with 1.00 respectively.

Familial risk was very high when both the mother and the sister were diagnosed with ovarian cancer, which may be related to high penetrant dominant effects. For the high-risk group of RR 10.40, histology was available only for ten patients; five were serous, two were non-specified adenocarcinomas and the remaining three were diverse histologies (clear cell, endometrioid and undifferentiated). Five of eight specific histologies were serous which may suggest an association with *BRCA1/2* [21, 22]. In a previous UK study, it was estimated that *BRCA1* and *BRCA2* mutations account for about 24% of the familial risk of epithelial ovarian cancer among first-degree relatives, and in the remaining cases familial relative risk was estimated at 2.24 [15]. That study reported a higher familial risk for serous than non-serous cases, most likely associated with *BRCA* mutations; in the present analyses no marked differences were noted. An unknown factor in population-based studies on ovarian cancer without data on mutation status is the lack of information on ovariectomies. Removal of ovaries in mutation carriers would obviously suppress familial risk for serous cancers.

Familial associations of ovarian cancer with histology- specific ovarian cancers are displayed in Table 3. Overall ovarian cancer risk was associated with family history of ovarian cancers of undifferentiated (4.79, *P* <0.001) > endometrioid (3.81, *P* <0.001) > sex cord-stromal (2.72) > mucinous (2.21, *P* <0.01) > clear cell (2.16) and > serous (2.15, *P* <0.001) type. On the other hand, i.e. for specific histological types of ovarian cancer when probands had any ovarian cancer, increased risks were found in all but mucinous and sex cord-stromal types. The order of RRs was undifferentiated (5.45, *P* <0.001) > serous (2.96, *P* <0.001) > endometrioid (2.81, *P* < 0.001) and > clear cell (1.67). In comparison, the international Ovarian Cancer Cohort Consortium found the overall familial risk was 1.48 in the combined cohort covering 5,584 invasive ovarian cancer patients and only serous ovarian cancer was observed to be associated with the family history of 1.61 [7]. The differences between the present familial risks (2.51, excess familial risk 1.51) and those reported by the Ovarian Cancer Cohort Consortium (1.48, excess risk 0.48) are vast. The authors of the international consortium did not compare their risk estimates to the reported values from the literature and we can only speculate that family history was underreported in that study [11–15]. Self-reported family histories tend to be unreliable and for ovarian cancer the positive predictive value of correct reporting was only 69% in a published pooled analysis [33]. Among epithelial ovarian cancers, serous, endometrioid and clear cell tumors are proposed to display Muellerian phenotype according to their origins [3]. Therefore, we did the similar analysis as Table 3 in S1 Table by only including undifferentiated, clear cell, endometrioid and serous types in overall ovarian cancers. However, no interesting results were found.

**Table 3:** Familial associations of overall ovarian cancer with histology-specific ovarian cancer.

Histological type	Overall risk of ovarian cancer in daughters				Risk of histology-specific ovarian cancer in daughters			
	*N1*	*N2*	*RR*	*95% CI*	*N1*	*N2*	*RR*	*95% CI*
Undifferentiated	8841	9	***4*.*79***	2.49–9.21	175	18	***5*.*45***	3.36–8.86
Clear cell	8843	7	**2.16**	1.03–4.54	496	15	**1.67**	1.00–2.80
Endometrioid	8821	29	***3*.*81***	2.65–5.49	951	48	***2*.*81***	2.10–3.75
Serous	8780	70	***2*.*15***	1.70–2.73	3934	215	***2*.*96***	2.58–3.40
Mucinous	8838	12	***2*.*21***	1.26–3.90	708	18	1.51	0.94–2.41
Sex cord-stromal	8846	4	**2.72**	1.02–7.25	295	5	1.10	0.46–2.66

N1: Number of cases without family history in first-degree relatives; N2: Number of cases with family history in first-degree relatives;

Bolding, italic and underlining indicate that the 95% CI, 99% CI and 99.9% CI did not overlap with 1.00 respectively;

Table 4 shows familial associations among different histological types of ovarian cancers in cases and probands and those associations are summarized in S2 Fig. Risk of undifferentiated ovarian cancer increased when a first-degree relative was diagnosed with clear cell (15.44, *P* <0.01), serous (6.01, *P* <0.001) and mucinous (9.23) ovarian cancer. Endometrioid ovarian cancer risk was associated with family history of the concordant histological type of ovarian cancer (3.59); increased risk of this histological type was also observed in family of patients affected by undifferentiated (9.27, *P* <0.01) and serous (2.26) ovarian cancer. With the exception of clear cell type, serous ovarian cancer risk was associated with family history of all the other histological types. Mucinous ovarian cancer risk was associated with the concordant histological type (6.91, *P* <0.001) and undifferentiated type (7.08). Risk of sex cord-stromal type increased in families of clear cell ovarian cancer patients (9.70). A striking finding was that concordant familial risks were highest only for mucinous cancer, for all others some discordant associations showed the highest RRs. For example, the endometrioid-undifferentiated RR was 9.27 and the serous-undifferentiated RR was 4.80. This suggests that histology in familial ovarian cancer is not specific, and if genes or polygenes contribute to familial clustering they may not define histology or that they are influenced by hormonal and environmental factors to a variable degree.

**Table 4:** Familial associations among different histological types of invasive ovarian cancers.

Histological types		Cases without family history	Cases with family history			
Offspring	First-degree relative		*N*	*RR*	*95%CI*	
					*Lower*	*Upper*
Undifferentiated	Clear cell	175	1	***15*.*44***	2.16	110.37
	Serous	175	4	***6*.*01***	2.23	16.2
	Mucinous	175	1	**9.23**	1.29	65.89
Endometrioid	Undifferentiated	951	2	***9*.*27***	2.31	37.12
	Endometrioid	951	3	**3.59**	1.15	11.14
	Serous	951	8	**2.26**	1.13	4.53
Serous	Undifferentiated	3934	4	***4*.*80***	1.80	12.8
	Clear cell	3934	3	2.08	0.67	6.45
	Endometrioid	3934	12	***3*.*50***	1.99	6.17
	Serous	3934	36	***2*.*47***	1.78	3.43
	Mucinous	3934	6	**2.44**	1.09	5.43
	Sex cord-stromal	3934	3	***4*.*62***	1.49	14.33
Mucinous	Undifferentiated	708	1	**7.08**	1.00	50.33
	Endometrioid	708	2	3.26	0.81	13.05
	Serous	708	4	1.56	0.58	4.17
	Mucinous	708	3	***6*.*91***	2.22	21.49
Sex cord-stromal	Clear cell	295	1	**9.70**	1.36	69.12
	Serous	295	2	1.97	0.49	7.93

Only items with at least two cases with family history, or with significant results are displayed in Table 4. No such items were found for clear cell type of ovarian cancer.

Bolding, italic and underlining indicate that the 95% CI, 99% CI and 99.9% CI did not overlap with 1.00 respectively.

The present study is by far the largest family study of its kind in the world published and one of the few studies reporting familial risks by specific histology in cases and probands. The main limitation of this study is that the cases with identifiable histology were diagnosed only after 1993 since the application of ICD-O/2 in the cancer registry. This affects familial risk estimates because 22 years of follow-up is short for intergenerational studies considering risks of both the parental and offspring generations. Furthermore, histological classification has not been updated to meet the current guidelines. For example, serous histology is now considered to be either low-grade or high-grade with different prognoses and molecular events/etiologies. Moreover, compared to low-grade serous ovarian cancer, high-grade serous, endometrioid, clear cell, mucinous types are considered to evolve from different pathways and originate outside of ovary: high-grade serous type may evolve from fallopian tube while endometroid and clear cell may originate from endometrial tissue passing through the fallopian tube resulting in endometriosis [3]. Insufficient clinico-behavioral information, such as smoking, is also a caveat in the analysis since they can be construed as potential confounders. However, as we adjusted the data for socioeconomic factors, this reduces greatly the possible confounding by smoking [34].

## Conclusions

In summary, by using the latest Swedish Family-Cancer Database, we found that each histological type of ovarian cancer was associated with at least two other histological types and was associated with the overall ovarian cancer, suggesting that the causes for familial clustering do not define a specific histology. Sharing of different histological types of ovarian cancer is likely an indication of the complexity of the underlying mechanisms. Our results provide useful information for genetic counseling; familial risks are high, particularly, for early-onset patients and for those with multiple affected relatives.

## Supporting information

S1 Fig Incidence by age group for overall and for different histological types of ovarian cancer in the period 1993–2015. (TIF)

S2 Fig Familial associations between different histological types of ovarian cancer. Risk of histology with grey background was significant within concordant histology of ovarian cancer. Risk of the two histologies between full line was significant in the two-way comparison. Risk of the two histologies between imaginary line was significant in one way and the histology the arrow points to is from offspring.
(TIF)

S1 Table Familial associations of overall ovarian cancer with histology-specific ovarian cancer by including undifferentiated, clear cell, endometrioid and serous types in overall ovarian cancers. (DOCX)
