# Correction: Familial risks of ovarian cancer by age at diagnosis, proband type and histology

**DOI:** 10.1371/journal.pone.0206721

**Published:** 2018-10-26

**Authors:** Guoqiao Zheng, Hongyao Yu, Anna Kanerva, Asta Försti, Kristina Sundquist, Kari Hemminki

Fig 1 is incorrect. Please view the corrected Fig 1 here.

**Fig 1:**
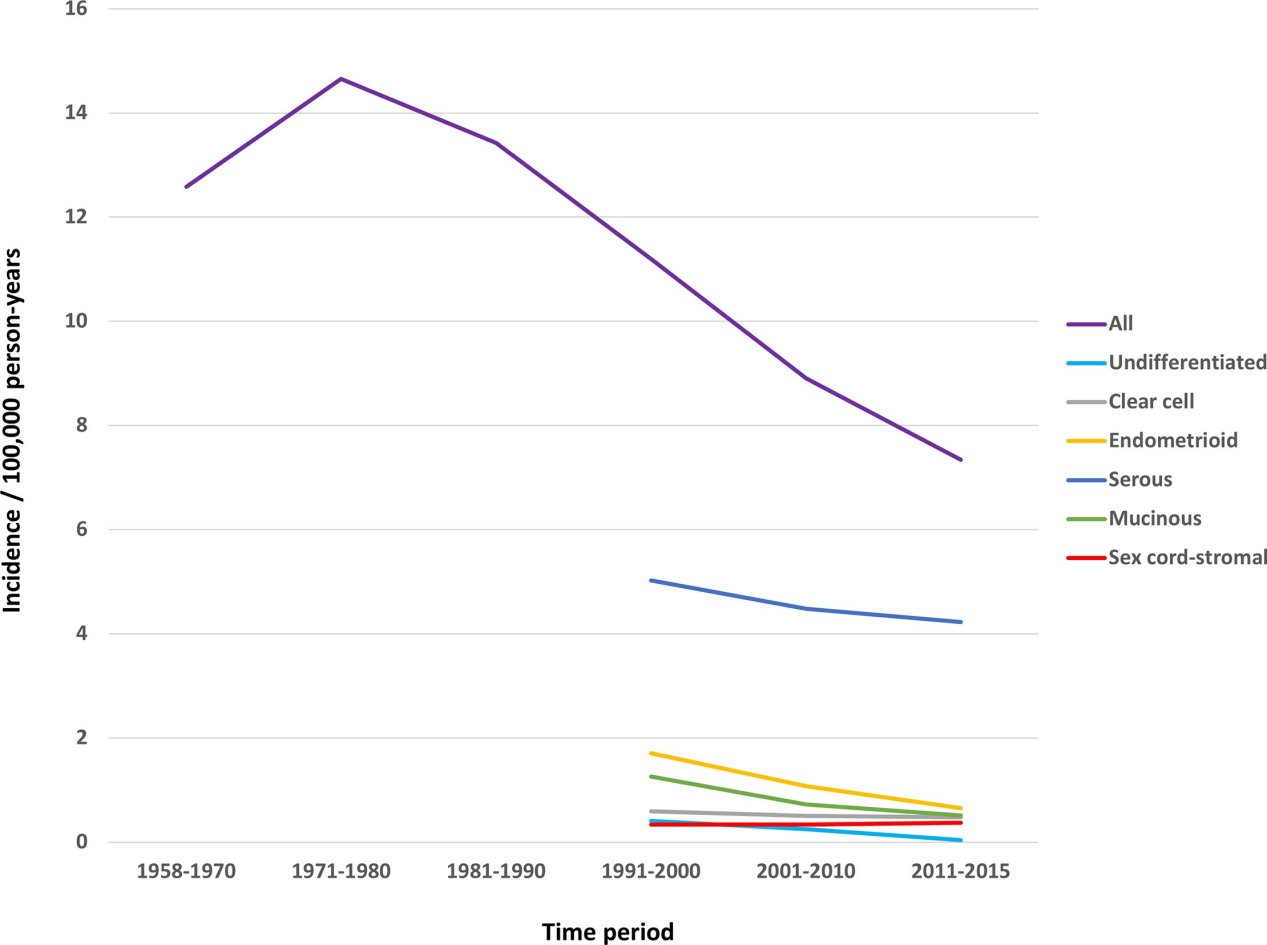
Age-standardized incidence in different time period for overall and eight histological types of invasive ovarian cancer. Since the record of SNOMED was started in 1993, the periods for subtypes only included 1993–2000, 2001–2010 and 2011–2015.
