# Supplementary figures and images for: Familial risks of ovarian cancer by age at diagnosis, proband type and histology
Source: PLoS One. 2018 Oct 3;13(10):e0205000. doi: 10.1371/journal.pone.0205000 (PMC6169923; doi:10.1371/journal.pone.0205000)

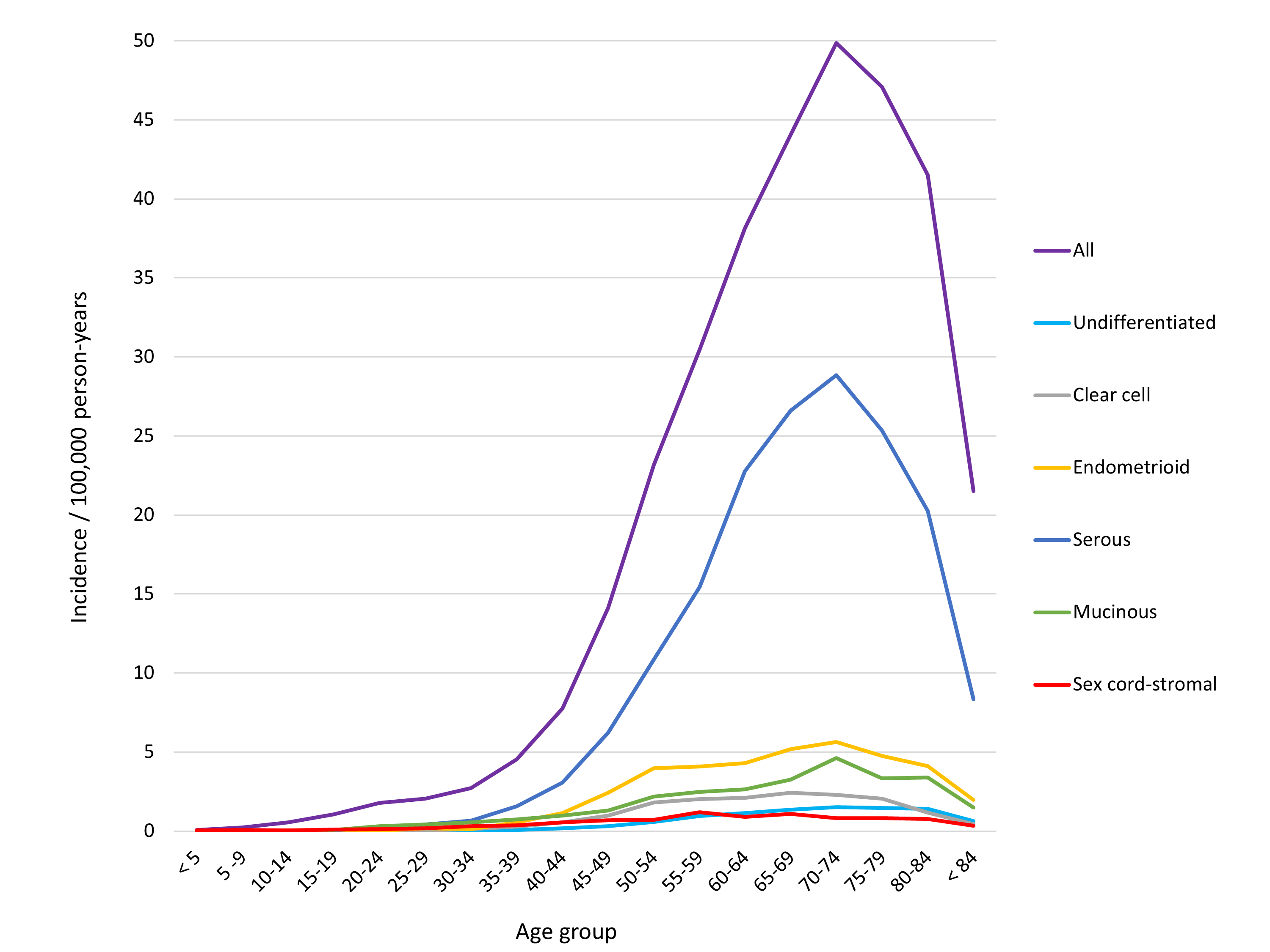

Supplement: S1 Fig — (TIF) [file pone.0205000.s001.tif]

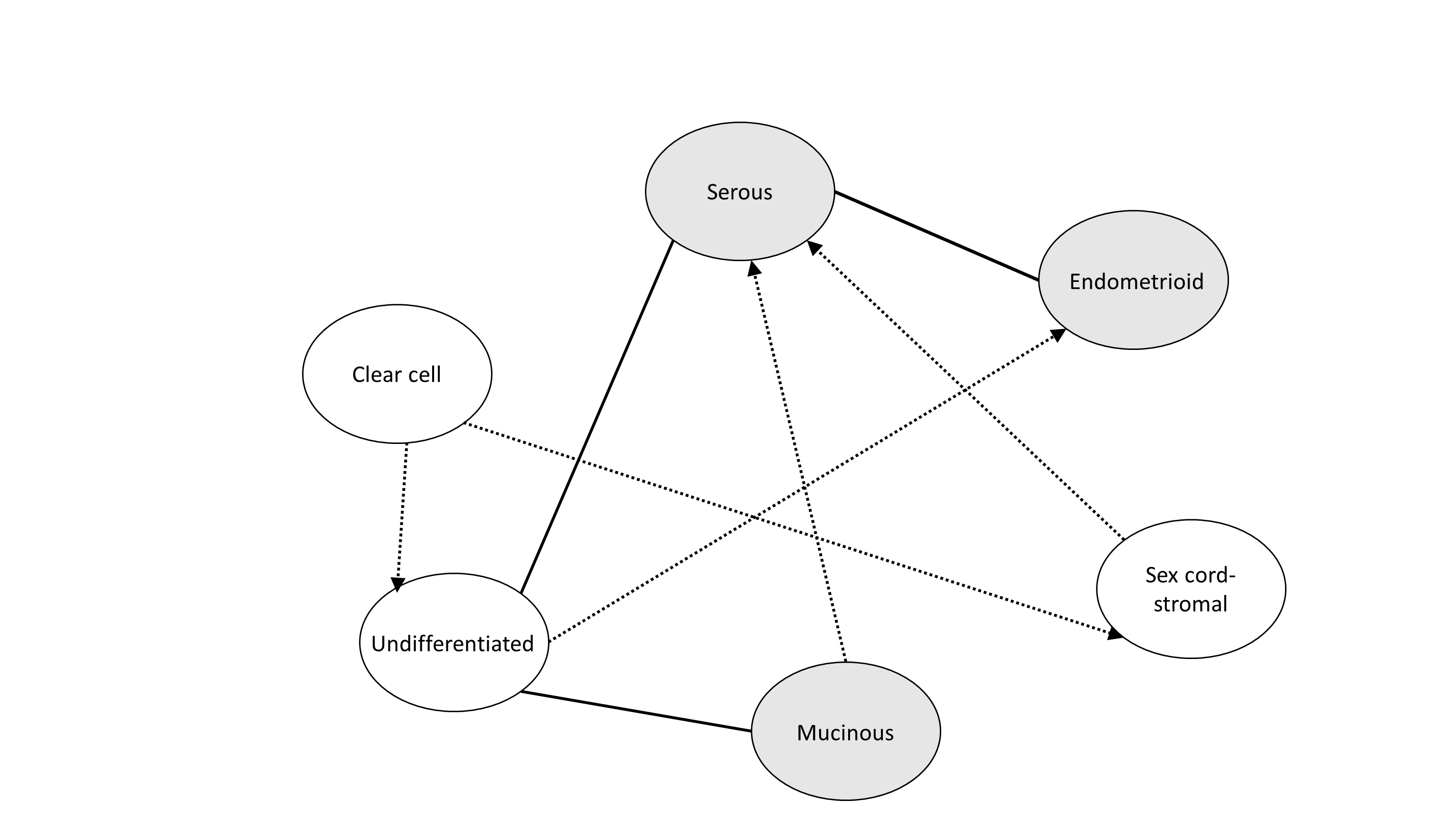

Supplement: S2 Fig — Risk of histology with grey background was significant within concordant histology of ovarian cancer. Risk of the two histologies between full line was significant in the two-way comparison. Risk of the two histologies between imaginary line was significant in one way and the histology the arrow points to is from offspring. (TIF) [file pone.0205000.s002.tif]
