# Supplementary material for: Familial risks of ovarian cancer by age at diagnosis, proband type and histology
Source: PLoS One. 2018 Oct 3;13(10):e0205000. doi: 10.1371/journal.pone.0205000 (PMC6169923; doi:10.1371/journal.pone.0205000)
Supplement: S1 Table — (DOCX) [file pone.0205000.s003.docx]

**S1 Table Familial associations of overall ovarian cancer with histology-specific ovarian cancer by including undifferentiated, clear cell, endometrioid and serous types in overall ovarian cancers**

| Histological type | Overall risk of ovarian cancer in daughters | | | | Risk of histology-specific ovarian cancer in daughters | | | |
| --- | --- | --- | --- | --- | --- | --- | --- | --- |
|  | *N1* | *N2* | *RR* | *95% CI* | *N1* | *N2* | *RR* | *95% CI* |
| Undifferentiated | 5845 | 7 | ***5.53*** | 2.64-11.61 | 175 | 6 | ***6.49*** | 2.88-14.65 |
| Clear cell | 5848 | 7 | 1.85 | 0.70-4.95 | 496 | 2 | 0.77 | 0.19-3.10 |
| Endometrioid | 5835 | 17 | ***3.32*** | 2.06-5.34 | 951 | 13 | ***2.63*** | 1.52-4.55 |
| Serous | 5798 | 54 | ***2.50*** | 1.92-3.72 | 3934 | 55 | ***2.70*** | 2.07-3.53 |

N1: Number of cases without family history in first-degree relatives; N2: Number of cases with family history in first-degree relatives;

Bolding, italic and underlining indicate that the 95% CI, 99% CI and 99.9% CI did not overlap with 1.00 respectively;
